# Comprehensive Review on Current and Future Regulatory Requirements on Wearable Sensors in Preclinical and Clinical Testing

**DOI:** 10.3389/fbioe.2019.00313

**Published:** 2019-11-08

**Authors:** Alice Ravizza, Carmelo De Maria, Licia Di Pietro, Federico Sternini, Alberto L. Audenino, Cristina Bignardi

**Affiliations:** ^1^Use-me-d S.r.l., Turin, Italy; ^2^Information Engineering Department, Research Center “Enrico Piaggio”, University of Pisa, Pisa, Italy; ^3^Polito^*BIO*^Med Lab, Department of Mechanical and Aerospace Engineering, Politecnico di Torino, Turin, Italy

**Keywords:** Medical Device Regulation, wearable medical sensor, medical device, accessory, component

## Abstract

Medical devices are designed, tested, and placed on the market in a highly regulated environment. Wearable sensors are crucial components of various medical devices: design and validation of wearable sensors, if managed according to international standards, can foster innovation while respecting regulatory requirements. The purpose of this paper is to review the upcoming European Union (EU) Medical Device Regulations 2017/745 and 2017/746, the current and future International Electrotechnical Commission (IEC) and International Organization for Standardization (ISO) standards that set methods for design and validation of medical devices, with a focus on wearable sensors. Risk classification according to the regulation is described. The international standards IEC 62304, IEC 60601, ISO 14971, and ISO 13485 are reviewed to define regulatory restrictions during design, pre-clinical validation and clinical validation of devices that include wearable sensors as crucial components. This paper is not about any specific innovation but it is a toolbox for interpreting current and future regulatory restrictions; an integrated method for design planning, validation and clinical testing is proposed. Application of this method to design wearable sensors should be evaluated in the future in order to assess its potentially positive impact to fostering innovation and to ensure timely development.

## Introduction

Medical devices are subject to strict controls and regulations all around the world. In Europe, any medical device shall follow a certification path that is designed to ensure its safety, efficacy, and constant quality level. If all regulatory requirements are satisfied, the device can obtain the CE Marking in Europe: it is common practice for the medical devices manufacturers to use harmonized standards to prove compliance to the relevant European legislation, which currently is composed of three European Council Directives and one Eudamed Commission Decision. In the next few years, from 2020 for medical devices and from 2022 for IVD, this legislation will be entirely repealed by entry into force of two regulations drafted in 2017, posing many challenges to the medical device manufacturers because of the burden of the regulatory framework.

This review, drafted after an in-depth analysis of new regulations and latest revisions of international standards, is intended to help the design of future medical devices ensuring compliance with new regulations, using relevant harmonized standards as part of the manufacturer's common practice, and it is oriented toward devices that include wearable sensors. While specific cases and discussions are proposed for wearable sensors, this analysis of requirements and standards is applicable to all medical devices. This review is focused on wearable sensors because they can be useful in medical devices of all kinds, providing real-life and real-time informations intended for monitoring, diagnosis and therapy too.

## Definition of Medical Device

Wearable health technologies offer great promise for reducing healthcare costs and improving patient care both in hospital and at home. Wearable technologies may perform various medical actions, including tracking, recording, and monitoring of biomedical signals. Wearable sensors include smart watches, patches, socks, and t-shirt, all characterized by the fact that they allow data transfer to another device (e.g., smartphone, cloud platform; Jeong et al., 2009; Kwak et al., 2011; Koydemir and Ozcan, 2018). Day by day, these technologies are becoming more popular among healthcare stakeholders. Associations for patient advocacy already consider them important tools to improve therapy involvement: but their widespread adoption is hindered by regulatory constraints that developers should, ideally, resolve in near future (Mosconi et al., 2019). These constraints are justified by the need of obtaining reliable, robust and safe data from a wearable sensor, if this is intended for a medical purpose and not only for a wellness purpose.

The European Union (EU) Medical Device Regulation (MDR) [Council Regulation 2017/745 (Regulation (EU) 2017/745, 2017)^1^ of April 5th 2017 concerning medical devices] repeals the existing directives on medical devices: Medical Device Directive 93/42/EEC (MDD) (Council Directive 93/42/EEC, 1993^2^) and Active Implantable Medical Device Directive 90/385/EEC (AIMD) (Council Directive 90/385/EEC, 1990^3^). The new regulation will enter in force on May 25th 2020 after a transition time of 3 years; it has a significant impact on manufacturers, due to an increased request of clinical data for assessment of the relationship between the clinical performance of the device and the consequent clinical benefit on the patient.

According to the EU MDR, the Medical Device definition is described below.

“*Medical device” means any instrument, apparatus, appliance, software, implant, reagent, material, or other article intended by the manufacturer to be used, alone or in combination, for human beings for one or more of the following specific medical purposes:*

- *diagnosis, prevention, monitoring, prediction, prognosis, treatment, or alleviation of disease*,
- *diagnosis, monitoring, treatment, alleviation of, or compensation for, an injury or disability*,
- *investigation, replacement, or modification of the anatomy or of a physiological or pathological process or state*,
- *providing information by means of in vitro examination of specimens derived from the human body, including organ, blood, and tissue donations*.

*and which does not achieve its principal intended action by pharmacological, immunological, or metabolic means, in or on the human body, but which may be assisted in its function by such means*.

The following products shall also be deemed to be medical devices:

- devices for the control or support of conception;
- *products specifically intended for the cleaning, disinfection or sterilization of devices as referred to in Article 1(4) and of those referred to in the first paragraph of this point*.

Wearable sensors may be used to collect physiological data, movement data, location data and others; the definition of medical device can of course interpreted for applicability to wearable sensors, when used with a medical purpose. The intended use defines if a wearable sensor is a medical device itself or is part of a medical device.

Moreover, the EU *in vitro* Medical Device Regulation (IVMDR) [Council Regulation 2017/746 (Regulation (EU) 2017/746, 2017^4^) of April 5th 2017 concerning *in vitro* medical devices] repeals the existing *in vitro* Diagnostics Directive 98/79/EC (IVD) and Eudamed Commission Decision 2010/227/EU (Directive 98/79/EC, 1998^5^; 2010/227/: Commission Decision, 2010). The new regulation will enter in force on May 25th 2022 after a transition time of 5 years; it has a significant impact on manufacturers, due to an increased request of performance data for assessment of the *in vitro* medical device.

According to the EU IVMDR, the *in vitro* Medical Device definition is described below.

“*In vitro diagnostic medical device” means any medical device which is a reagent, reagent product, calibrator, control material, kit, instrument, apparatus, piece of equipment, software or system, whether used alone or in combination, intended by the manufacturer to be used in vitro for the examination of specimens, including blood and tissue donations, derived from the human body, solely or principally for the purpose of providing information on one or more of the following:*

- concerning a physiological or pathological process or state;
- concerning congenital physical or mental impairments;
- concerning the predisposition to a medical condition or a disease;
- to determine the safety and compatibility with potential recipients;
- to predict treatment response or reactions;
- *to define or monitoring therapeutic measures*.
- *Specimen receptacles shall also be deemed to be in vitro diagnostic medical devices*.

Wearable sensors (for example biosensors for monitoring of clinically relevant biomolecules for diagnostics), may be used to collect specimens from the human body, and to provide information for diagnostic, monitoring or compatibility purposes: The intended use defines if a wearable sensor is an *in vitro* medical device itself or is part of an *in vitro* medical device.

## Definition of Component of Medical Device and of Accessory of Medical Device; Their Classification

The current MDD-AIMD directives (Council Directive 93/42/EEC, 1993^2^; Council Directive 90/385/EEC, 1990^3^) and the upcoming MDR (Regulation (EU) 2017/745, 2017^1^) regulation provide significant information regarding parts of a finished medical device and accessories. These comments are also valid for *in vitro* medical devices.

So, “accessory for a medical device” means an article which, whilst not being itself a medical device, is intended by its manufacturer to be used together with a medical device to specifically enable the medical device to be used in accordance with its intended purpose or to specifically and directly assist the medical functionality of the medical device.

This definition may well be applied to a wearable sensor which may not by itself be a medical device with a well-defined medical purpose, but that is used to collect information that is used as input data for subsequent diagnosis or therapy management by a medical device or by a medical-grade software. Similarly, this definition can be applied to a chemical biosensor that collects biomolecules and then forwards the collected information for diagnostic purposes.

A good example may be an actigraph unit that provides information to a medical app, that monitors the sleep/wake cycle to diagnose insomnia or the level of activity (De Leonardis et al., 2018). For *in vitro*, a good example may be a wearable biosensor designed for the monitoring of analytes contained in sweat, which sends the raw data to devices with proper computational power to complete further analyses (Sempionatto et al., 2017).

Accessories are products in their own right and, as a general rule, do not follow the classification of related devices in conjunction with which they are used. Accessories are therefore following both current MDD-AIMD directives (Council Directive 93/42/EEC, 1993; Council Directive 90/385/EEC, 1990^3^) and upcoming MDR (Regulation (EU) 2017/745, 2017^1^) and IVMDR (Regulation (EU) 2017/746, 2017^4^) to be classified in their own right.

This implies that, in case of wearable sensors, they will most probably fall in either class IIa or class IIb of the upcoming MDR (Regulation (EU) 2017/745, 2017^1^). In some cases, the sensors may be part of a class III system. The potentially adequate rules may be:

- for wearable sensors intended for monitoring of the patients' parameters: rule 10, that places active devices for monitoring in class IIa as a general rule and in class IIb in case of monitoring of “vital parameters in dangerous situations;”
- for wearable sensors that monitor or manage the performance of a device: rule 9, that classifies these devices in class IIb or class III if the influenced device is implantable;
- for wearable sensors that are part of closed loop controllers: rule 22 places devices in class III if they have a “diagnostic function which significantly determines the patient management by the device;” else class IIb as per rule 9.

Similarly, for IVMDR the classification rules are proposed according to a risk based approach and the risk classification is based on the kind of diagnosed state. For example, the highest class D is assigned to *in vitro* devices intended to diagnose transmissible agents that can pose a risk to the public health (i.e., in donated blood for transfusion, or for diseases with high propagation potential). All devices that are intended for self testing fall in class C, unless they diagnose states such as pregnancy and fertility or some urine indicators. It is most probable that many biochemical sensors will be classified in class C as *in vitro* self-test devices.

## Certification Path for Wearable Sensors in Europe

In some cases, commercial wearable sensors are used in applications that, while resembling a medical application, do not actually provide a measurable clinical benefit for the patient and that are usually defined as “wellness,” “self-enhancement,” or “self-tracking.” These devices are not designed and validated as medical devices, since their intended use cannot be included in any of the medical purposes above. So, these devices are designed to be electrically safe but they have no data integrity and accuracy requirements other than commercial expectations and they do not fall under the MDD-AIMD directives (Council Directive 90/385/EEC, 1990^3^; Council Directive 93/42/EEC, 1993^2^) and the upcoming MDR (Regulation (EU) 2017/745, 2017^1^) requirements.

If data from a commercial wearable sensor are later used as inputs to a medical device, for example if these data are used as input data for a diagnosis software, the manufacturer of the medical device is using the data in an unforeseen use of the sensor. In this case the end user of the data, not the supplier for the wearable sensor, shall prove that the quality of the data collected from the sensor is adequate for a medical grade use. Medical device designers may approach this aspect by treating the commercial (non-medical) wearable sensor as a black box. This approach presents multiple difficulties, both legal and technical, and should be discouraged wherever possible.

Medical devices in the EU are subject to the MDD-AIMD directives (Council Directive 90/385/EEC, 1990^3^; Council Directive 93/42/EEC, 1993^2^) and the upcoming MDR (Regulation (EU) 2017/745, 2017^1^) from the pre-market phase of clinical testing and during all their life cycle. For the premarket phase, the safety and traceability requirements apply, together with ethics requirements that protect the participants of the clinical trial. For the market phase, manufacturers must place a CE marking on the product. CE marking indicates that the medical device complies with the applicable EU regulations and enables the commercialization of the products in all European countries.

The organization that is sponsoring the clinical trial on patients and later is placing the device on the market is responsible for maintaining regulatory compliance, regardless of whether they outsource any or all steps of the development and manufacturing operations.

**Figure 1:**
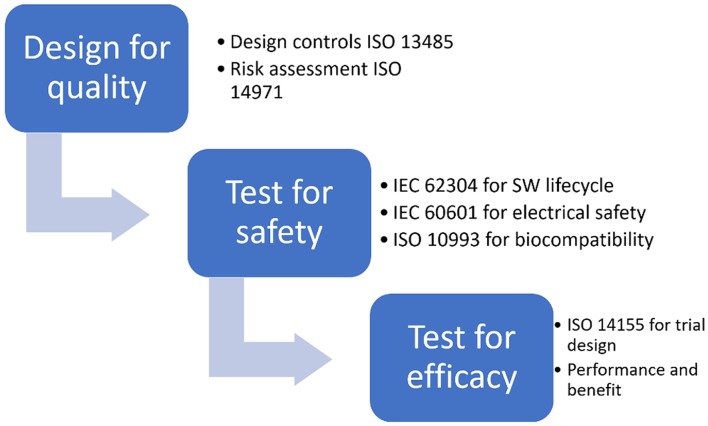
Risk based approach for CE marking.

CE marking is obtained, for all classes of medical devices except class I, only after an EU-approved organization (called Notified Body) has verified that the product complies with all regulatory requirements. The scheme of the risk based approach to correctly design a medical device and prove its adequacy to regulatory requirements, thus obtaining CE marking, is shown in Figure 1.

## Wearable Sensors for Monitoring, for Feedback Control, for Specimen Diagnosis

As said wearable sensors can be used in medical applications intended to treat diseases and impairments, to monitor different conditions and physiological parameters, to evaluate specimens from the human body.

A well-known example of a wearable sensor for monitoring is the photoplethysmograph (PPG), used to measure heart rate, by the assessment of a variation of intensity of reflected fraction LED-emitted light, which is correlated with the volume of blood in the illuminated tissue. Recent studies could lead to remote measurements of heart rate variability, using PPG (Qureshi and Krishnan, 2018). Investigation of vascular conditions is crucial for population health and research led to continuous monitoring of cardiovascular system using innovative techniques like auscultation of heart sounds and low energy electromagnetic radiofrequency waves (Amir et al., 2016; Qureshi and Krishnan, 2018).

In motion analysis, wearable devices are used mainly to monitor activity status, which is fundamental in rehabilitation phase, to perform gait analysis and to recognize activities, anomalies and accidents (Appelboom et al., 2015; Agostini et al., 2017). The technology of these devices is usually similar to commercial trackers, with multiple integrated sensors: accelerometers, used to measure accelerations and forces (Menicucci et al., 2006; Zanetti et al., 2013; Schierano et al., 2016); magnetometers, designed to detect device orientation; gyroscopes, which give rotational information. State of the art systems include all three, obtaining 9° of Freedom measurements (acceleration along three axes, rotation rate around three axes and three-component magnetic field intensity; Qureshi and Krishnan, 2018). Output of these sensors and of possible additional sensors (e.g., altimeter, barometer) are used to enhance knowledge about the medical tasks performed by the patient.

Wearable devices can also be used to manage systems, for example sensors used to control orthoses, prostheses (Zanetti et al., 2018a,b) and tele rehabilitation robots, but different technologies are required. The system needs information to plan and control its subsequent actions; the sensor decision strategy can be different. In case of orthoses and prostheses the main solutions are wearable surface electromyograph (sEMG). The sensor is composed by dry skin electrodes to collect the electric signal, conditioning circuit, and data processing unit. Using myoelectric signals in clinical decision making is still a challenge, but machine learning approaches showed that it is possible to control prostheses using electromyography (EMG) (Castellini and van der Smagt, 2009).

In rehabilitation systems, the wearable sensor shall give precise, continuous and reliable information about patient limb position, especially if the device is providing information to a rehabilitation robot that mobilize patient limbs. For this reason, all the solutions described above can be inadequate. At the current state of knowledge, the main solution is the integration of an accelerometer and a gyroscope with kinematic models, in order to obtain the needed accuracy, but future development in nanosensors and textile electronics could lead to innovative stretch and angle sensors (Šlajpah et al., 2014).

Lastly, biochemical sensors are mainly used to monitor chemical or biological parameters of body fluids, to discover novel biomarkers and to study the biochemical composition of body fluids. Now the most common application for both research and commercial device is the glucose monitoring, but various proof of concept demonstrations of non-invasive or minimally invasive devices have been already applied to many applications detecting different analytes in easily accessible fluids like saliva, sweat, and interstitial fluid (Kim et al., 2019).

The adequateness of the level of accuracy of these systems is not pre-determined by the regulation or by an International Organization for Standardization (ISO) standard, but it shall be evaluated for each medical purpose. In case of activity/pattern recognition the accuracy shall be evaluated by comparing the classification output with ground truth obtained via state-of-the-art methods or with pre-classified data. On the other hand, if the system output is a measurement, the accuracy shall be assessed evaluating the measurement against a primary instrument. In certain cases, the primary instrument can be a state-of-the art medical device with a measuring function.

In case of wearable sensors intended to provide significant information to feedback loop controlled complex system, the accuracy shall be assessed not only on the output of the sensor, but also on the output of modules which process the sensor measurement and then reacts to the acquired information.

In case of sensors intended for *in vitro* diagnosis, the performance is assessed by the capability of the sensor “*to correctly detect or measure a particular analyte”* as for the IVMDR.

## Brief Introduction to Medical Device Regulation (MDR): Requirements on Safety, Performance, and Benefit, Consistent Level of Quality

All medical devices, including accessories, must comply with the Essential Requirements of the current MDD-AIMD (Council Directive 90/385/EEC, 1990^3^; Council Directive 93/42/EEC, 1993^2^), which are also a core requirement in the new MDR (Regulation (EU) 2017/745, 2017^1^), with significant changes and restrictive requirements, especially regarding the obligations of demonstration of clinical benefit. The obligations of the manufacturers can be summarized with three key words: safety, benefit, quality. International standards are adequate tools to provide proof of compliance to these core requirements.

For the demonstration of safety, developers of wearable sensors cannot derogate from the application of the ISO 14971 (British Standards Institution, 2012) standard, related to risk management, which impacts in particular the design and testing phases of development. Application of this standard proves that the wearable sensor is free of structural defects that could pose a risk for the patient or compromise the correct functioning, expressed in terms of technical performance.

The ISO 14971 standard presents, in annex C, a list of questions that may be used to identify the device characteristics that are more related to safety in any medical device. Amongst the questions, some are typically applicable to wearable devices:

### C.2.11. Are Measurements Taken?

In case of wearable sensors, the device typically measures physiological parameters such as movement, pressure, vibration, color shades. Else, they measure analytes in specimens from breath, spit, sweat, blood, and more. A combination of wearable sensors increases the amount of information: for example, a time stamp of each event, the orientation in space of the wearable device, absolute location of the sensor, proximity to objects or other sensors, environmental information like temperature, humidity, environmental light, altitude etcetera.

In order to describe risk correctly, developers should be able to define, for each of these parameters, the expected level of sensitivity and specificity of detection and relate the non-compliance to such level of quality to any hazard that may affect the patient.

Developers can explore the relationship between the device performance and the risk for the patient health, by asking questions like: If the wearable device is not accurate enough, will the patient be actively damaged? Will the patient be diagnosed or cured with a delay?

The answer to these questions shall consider device performance and intended use. If the device measures physiological parameters to define or influence the therapy, it may present a high-risk profile. In fact, an incorrect measurement can lead to incorrect therapy. If the device is used to directly diagnose a disease or condition the risk profile shall be similar to the case of device that directly control therapy, since the data are not controlled by the physician. If the device is used only to monitor a condition or to inform the diagnosis, the possible error is not automatically propagated to the therapy, leading to a lower risk profile.

### C.2.15 Is the Medical Device Susceptible to Environmental Influences?

In case of wearable sensors, they may be susceptible to specific conditions of the external environment, that impact the basic principles of functioning. Typically, they may be susceptible to vibrations, other environmental influences as humidity and temperature or may need protection from dust and water. In case of motion sensors, the devices can be influenced by environmental electromagnetic fields, since most of device use magnetic field information to extract the device orientation in space. This factor shall be considered especially if the device is intended to be used in a clinical institution, where machines like MRI devices and other electromedical devices can influence the magnetic field.

### C.2.26 Does Installation or Use of the Medical Device Require Special Training or Special Skills?

Wearable sensors may require different skills. As part of the usability evaluation, test scenarios regarding hazardous use may enlighten the possibility of improper or impaired use by patients with disabilities, too young or too old, that don't use the wearable device for as long as needed to obtain a good amount of data, and so on.

In many cases, a dedicated training may be required for particular wearable devices or for populations with a low level of medical literacy.

### C.2.29 Is Successful Application of the Medical Device Critically Dependent on Human Factors Such as the User Interface?

Significant factors may include: the case, for the handling and grasping of device. Developers should ask define impacts on use experience in various scenarios. For example, if the sensor is part of a bracelet or another object that is usually worn for beauty or if the sensor is supposed to be invisible, sticking on the skin.

Indicators for light, audio, haptic feedback should also be considered in their capability to enhance the user experience; on the other hand, they pose the potential risk of delivering unclear information to the end user.

## The Relationship Between Performance and Benefit

In medical devices, an adequate technical performance is the first step to the obtainment of a clinical benefit. In case of wearable sensors intended to measure physiological parameters, there is a clear relationship between accuracy and clinical benefit, since devices that provide information to ease diagnosis and monitoring rely on the quality of data. On the other hand, a wearable sensor can also provide other benefits: for example, it can be less obtrusive in the patients' life, improving general quality of life. Additionally, motion and gait analysis sensors can provide real life information, typically more reliable than the information gathered in a simulated environment to assess patient conditions. Moreover, collecting information that not accessible with other methods, like behavioral and motion data, enhances and increases efficiency of the evaluation of clinical conditions (Gresham et al., 2018). For these reasons, once the wearable sensor has been appropriately calibrated to match state-of-the -art accuracy, the relationship between technical performance and clinical benefit is well-described by indicators about quality of life and ergonomics.

In case of wearable sensors intended to provide information for a feedback loop complex system, the clinical benefit to the patient is provided not by the sensor itself but by the output of the complete system as a whole. The sensor accuracy can so directly improve clinical benefit, since it defines the quality of the input in a feedback system which will use the sensor information to plan and complete a therapy. For example, in a tremor reducing system the device needs an adequate input information to properly distinguish voluntary movement from tremor. So, the high accuracy of the sensor allows to properly reduce the tremor intensity while not suppressing volitional movement (Herrnstadt et al., 2019).

In case of a wearable sensor that is intended to detect an analyte with a reliable accurateness, the clinical benefit is provided by the subsequent use of the information provided by the sensor. If it is used to diagnose a disease, monitor a condition, or to detect a biomarker, the clinical benefit can be easily associated with the quality of the measurement, while if it is used to control and monitor a therapy, the clinical benefit is provided by the improvement of the therapy itself, whether the interpretation of sensor measurement and consequent therapy modifications are completed by a device, a physician or the patient. Moreover, if the sensor replaces an invasive or uncomfortable device, it will improve the quality of life of the patient. For example, the use of continuous glucose monitors can provide clinical benefit to patients with diabetes reducing glycated hemoglobin and the time spent in hypoglycemic condition (Floyd et al., 2012).

## Giving Proof of Safety of a Wearable Sensor

Proof of safety is achieved by a three steps approach: identify test requirements and methods, test for compliance, assess the test results to understand if risk minimization is acceptable.

Once the standard ISO 14971 has been used to identify the hazardous situations and the international standards have been searched for appropriate safety requirements, a complete set of tests for safety should be available. Prototypes sent to testing shall be significant of the final product.

For wearable sensors, the main risks may be summarized as follows, and presented in an order that respects the highest estimated burden to the developers:

- Software malfunction. Software malfunction, in terms of bugs, loss of data and/or inadequate performance is a key risk to be taken into consideration. The non-availability of the information provided by the wearable sensor may lead to various levels of consequences, from simple frustration to actual loss of operation of a life-saving device. In all wearable devices, and particularly in the ones used as part of closed loop controllers, software must be designed methodically and validated comprehensively. The standard IEC (International Electrotechnical Commission) 62304 (International Electrotechnical Commission; International Organization for Standardization, 2015) provides guidance and it should be noted that this standard is still in the 2006 edition at the time we are writing this article, even if a significant draft is available for consultation.
- Hackering of data: clinical data are precious, non-tangible assets. Special regulations apply to the protection of medical device under a cybersecurity point of view. Detailed guidelines are available from FDA (Food and Drug Administration) in both the premarket (U.S. Food Drug Administration., 2018) and post-market phase (U.S. Food & Drug Administration, 2016). It should be noted that the available international standard, while being widely applied, presents significant gaps in the definition of requirements (Anderson and Williams, 2018).
- Data transfer: the loss or alteration of data may impair the use of the whole wearable device. In particular the developer shall consider this main risk during the design of the device, especially during the communicating protocol definition, which can be oriented toward energy optimization but shall guarantee data integrity.
- Electrical hazard and Electromagnetic compatibility: this is particularly applicable for wearable devices that can be classified as “applied parts” of an electro-medical device. In the state-of-the-art, a very simple example of an “applied parts” of an electro-medical device is an electrode that is physically connected to an ECG (electrocardiogram) monitor. For wearable devices, an “applied part” could be a bracelet or even an electrode. These requirements are met by designing the hardware components as per IEC 60601 (International Electrotechnical Commission, 2012). It should be noted that this standard has a significant number of ancillary documents: the most appropriate to wearable sensors will be IEC 60601-1-2 regarding electro-magnetic compatibility and most probably also IEC 60601-1-6 regarding usability, IEC 60601-1-8 regarding alarms and indicators, IEC 60601-1-11 regarding devices to be used in home settings. For wearable sensors intended to be part of medical devices that are used in closed loop systems, the IEC 60601-1-10 is a core requirement standard. This standard requires that such systems are be stable, reliable, and fault tolerant.
- Biocompatibility. Most wearable sensors simply come in contact with intact skin. Regardless the very low level of biocompatibility risk, irritation, and allergic reactions should be taken into account. The ISO 10993-1 standard (International Organization for Standardization, 2018) provides complete guidance: appropriate testing may involve material characterization and, in some cases, some *in vitro* tests. Tests for toxicity of components of the wearable sensor may be needed in case of suspected exposure, in this case the ISO 10993-12,−17, and – 18 provide test methods for detection of compounds.
- Physico-Chemical stability: in case of biosensors, it may be adequate to evaluate the impact of environmental influences (heat, humidity, radiation) on the sensor performance. This is typically achieved by dedicated challenge tests.

## Giving Proof of Performance of a Wearable Sensor: Input Requirements, Design Outputs, and Verification as Per ISO 13485

ISO 13485 (International Organization for Standardization, 2016) is a widely applied standard that provides not only guidance on good manufacturing practices, but also guidance on design control for medical devices.

Design control begins with the clear identification of input requirements. Inputs include functional and performance requirements, user needs in terms of ergonomics and of expected benefit, and the identification of those main risks that shall be solved with safe-by-design solutions. Additionally, regulatory constraints are defined.

For wearable sensors, adequate input requirements may include: accuracy and robustness of the measurements taken, data transfer policies and standards, choice of adequate power source and power time duration, choice of ergonomic features, (for example in terms of shape, weight, aspect and adherence to the skin). Moreover, inputs shall include the definition of data security risks and of electrical risks that need a safe-by-design approach. Regulatory constraints may include restrictions on the choice of materials, of suppliers of parts and on use of open source software.

Design control continues in the design output phase, where multiple options and multiple prototypes are developed and tested against the requirements, to reach a proof of concept prototype and, in later phases, a significant commercial prototype. Design control shall document the different iterations and justify the choices that led to define which was the most adequate, amongst all possible variants.

Later, a significant prototype is subject to regulatory verification and validation, in terms of safety testing and in terms of performance testing. During this phase, the input requirements shall be reviewed and the tests shall give proof that they have been met. For example, software verification according to IEC 62304 may be used to give proof of software consistent quality, usability testing on real users may give proof of correct ergonomics, accuracy and repeatability testing may give proof of adequate performance.

## Minimum Requirements to Access to the Clinical Phase: ISO 14155

For demonstration of benefit, developers may refer to the ISO 14155 standard (International Organization for Standardization, 2011), related to good clinical practices for trials of medical devices on human patients.

The result of applying this standard demonstrates that there is a statistically significant relationship between the appropriate technical performance of the device and the actual clinical benefit to the patient.

When they test the device on human beings, being patients or healthy volunteers, in order to collect information regarding the expected clinical benefit, the developers shall respect legal and regulatory requirements that ensure the highest level of protection of the human being.

Trials that are intended to give proof of clinical benefit shall receive a preliminary approval of an Ethics committee: the approval is based on the review of the risk profile of the device, including adequacy for electrical safety and software reliability. Additionally, the ethics review will ensure that an adequate amount of data is collected for statistically significant results.

For this reason, the minimum requirements to obtain approval include at least giving proof of the device safety and giving proof that, for each participant to the trial, the participation to the study represents a potential beneficial impact on his/her clinical condition.

According to ISO 14155, the principal investigator of the study and the Ethics Committee shall receive a document (investigator brochure) that summarizes the risk- benefit profile of the device under assessment. Part of this document is the proof of safety according to harmonized standards (typically, at least electrical safety as per IEC 60601-1). Additionally, other safety testing may be required: for example, biocompatibility as per ISO 10993 or proof of software verification as per IEC 62304.

## Giving Proof of Clinical Benefit: Pilot and Pivotal Clinical Studies as Per ISO 14155

It is no longer sufficient to demonstrate that the wearable sensor “works”: accuracy and sensitivity are just the core requirements for the technical performance.

To use the wearable sensor in a clinical setting, developers should also give proof that it obtains, in a statistically significant way, the clinical benefit for which it was designed. In many cases, it will not be possible to gather sufficient clinical evidence in the literature and it will be necessary to perform appropriate clinical studies (Regulation (EU) 2017/745, 2017^1^).

Designers may refer to the ISO 14155 standard, which provides methods for planning and monitoring the study.

It is important to note that the clinical benefit can be expressed either as an in impact on health (or quality of life) of the individual patient or as an impact on the management of the therapeutic path through HTA (Health technology Assessment) indicators.

Firstly, the clinical evaluation is focused on the relationship between technical indicators and clinical indicators. Typically, this relationship is studied in small studies, although adequate for a good statistical evaluation. These studies should include both safety endpoints (often also measured in terms of technical malfunctions) and technical performance endpoints, while clinical efficacy endpoints are hypothesized and tested.

A “pilot” study is normally described as a small-scale study, useful to verify if the project is adequate, to establish its feasibility or to obtain information that allows to determine the size of the sample of the definitive study. A pilot study, in the case of medical software, may be relevant in case of:

- Need to study usability and “wear-ability” of the sensors.
- Need to better define the target population or the time schedule of the study monitoring visits.

In pilot studies, although statistical analyzes are still relevant, there is ample flexibility in the design of the study: therefore, often non-randomized studies without control will still be adequate (Thabane et al., 2010; Boudard et al., 2013). Furthermore, efficacy endpoints will not always be required, while safety endpoints can not be overlooked (Downey et al., 2018).

A “pivotal” study is instead considered a specific study to provide the data necessary for a regulatory approval. A regulatory study aims to gather clinical evidence to confirm that the benefit-risk ratio of the medical device is favorable to the patient. A pivotal study, in the case of wearable sensors for medical monitoring or feedback, must be performed in all cases where the evidence in the literature is not sufficient for CE certification (Regulation (EU) 2017/745, 2017^1^).

In this case, it will be important for the study design to collect statistically significant evidence. A double blind, randomized study may be difficult to design in case of wearable sensors, which makes the choice of endpoints and metrics even more important.

## Conclusions

The modifications to the European legislation brings many issues in the regulatory path for medical devices, that can be tackled quickly with a structured design plan that follows regulatory requirements step by step as illustrated in this review. We propose a strategy that allows to obtain a safe and reliable medical device by controlling the realization of the device from the prior risk analysis to the clinical benefit demonstration. The pivot of the device design is the definition of the use destination and all subsequent analysis including the definition of the main risks for the patients. Wearable sensors have many advantages in clinical practice, as discussed above, for example they are useful in improving data gathering and patient quality of life. To maintain such promise wearable shall be reliable and safe and for this reason their design shall consider many requirements.

## Author Contributions

AR and CD: conceptualization. AR: methodology. LD and FS: resources. AR and FS: writing—original draft preparation. CB and AA: supervision.

### Conflict of Interest

AR was employed by company Use-me-d S.r.l. The remaining authors declare that the research was conducted in the absence of any commercial or financial relationships that could be construed as a potential conflict of interest.

## Footnotes

^1^Regulation (EU) 2017/745 of the European Parliament and of the Council of 5 April 2017 on medical devices, amending Directive 2001/83/EC, Regulation (EC) No 178/2002 and Regulation (EC) No 1223/2009 and repealing Council Directives 90/385/EEC and 93/42/EEC (Text with EEA relevance.). 117.

^2^*Council Directive 93/42/EEC of 14 June 1993 concerning medical devices, 1993, Vol*. OJ L.

^3^*Council Directive 90/385/EEC of 20 June 1990 on the approximation of the laws of the Member States relating to active implantable medical devices, 1990, Vol*. OJ L.

^4^Regulation (EU) 2017/746 of the European Parliament and of the Council of 5 April 2017 on *in vitro* diagnostic medical devices and repealing Directive 98/79/EC and Commission Decision 2010/227/EU (Text with EEA relevance*)*, 117.

^5^*Directive 98/79/EC of the European Parliament and of the Council of 27 October 1998 on in vitro Diagnostic medical devices, 1998, Vol*. OJ L.
